# Supplementary figures and images for: Human herpesvirus 6B glycoprotein B postfusion structure, vulnerability mapping, and receptor recognition
Source: PLoS Pathog. 2025 Jul 9;21(7):e1013300. doi: 10.1371/journal.ppat.1013300 (PMC12240383; doi:10.1371/journal.ppat.1013300)

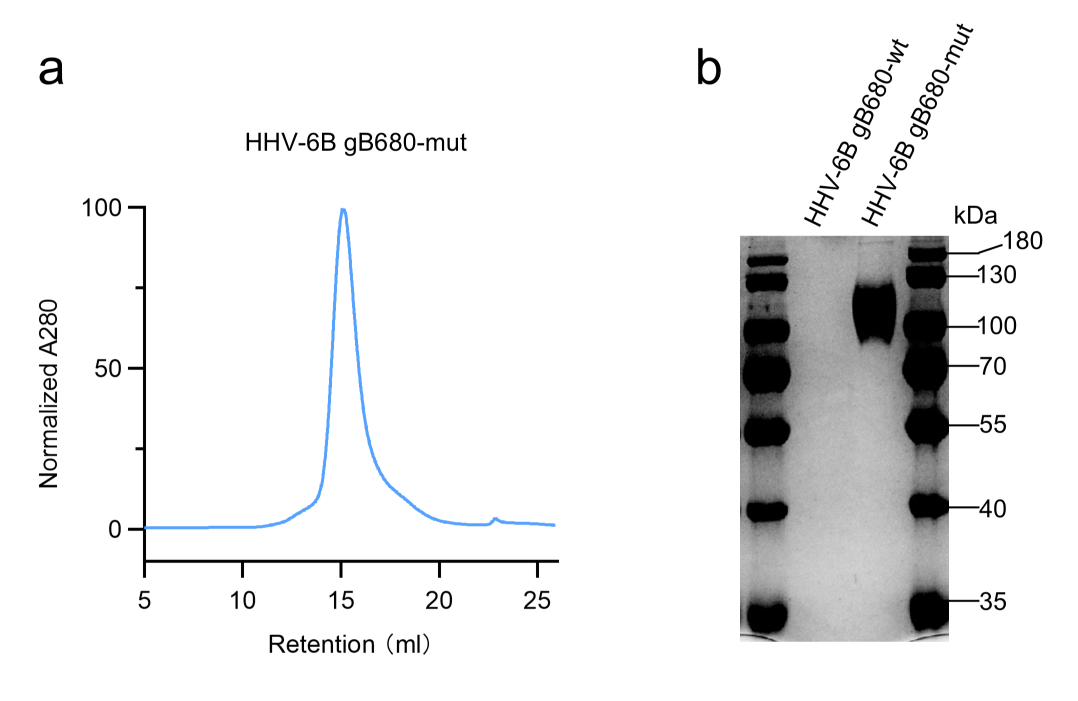

Supplement: S1 Fig — a, Size-exclusion chromatography (SEC) profile for HHV-6B gB680-mut ectodomain, performed on a Superdex 200 Increase 10/300 GL column. b, SDS-PAGE analysis showing the purification results for both wild-type HHV-6B gB680 (HHV-6B gB680-wt) and the mutant construct (HHV-6B gB680-mut). (TIF) [file ppat.1013300.s001.tif]

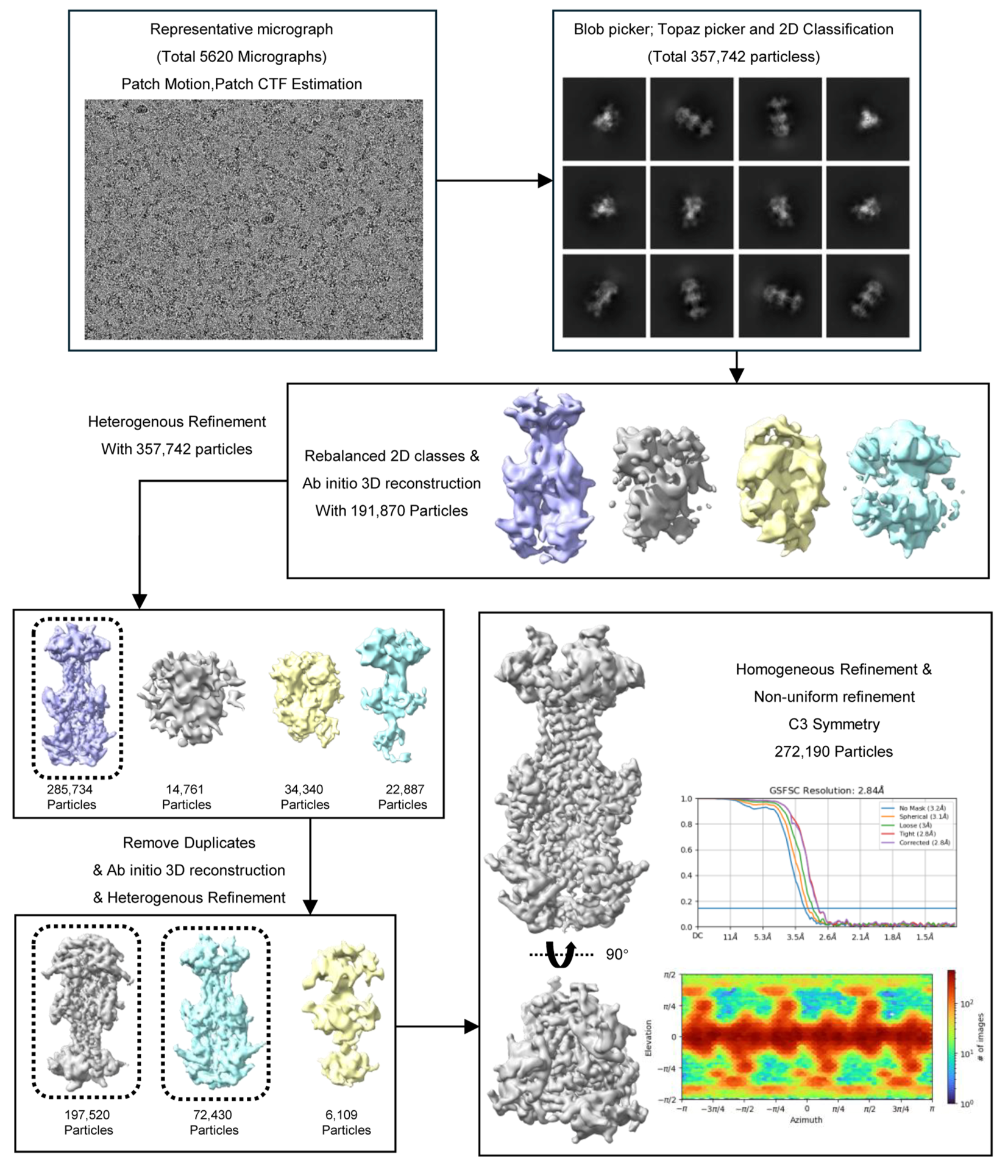

Supplement: S2 Fig — The workflow includes motion correction, contrast transfer function (CTF) estimation, particle picking, 2D classification, ab initio 3D reconstruction, and iterative heterogenous refinement steps. The Gold-standard Fourier shell correlation curve for the HHV-6B gB ectodomain. The Gold-standard Fourier shell correlation (FSC) curve for the HHV-6B gB ectodomain indicates a final refined resolution of 3.0 Å. The structure model is presented at the bottom of the figure, with both the FSC curve and the map of orientational distribution displayed to its right. (TIF) [file ppat.1013300.s002.tif]

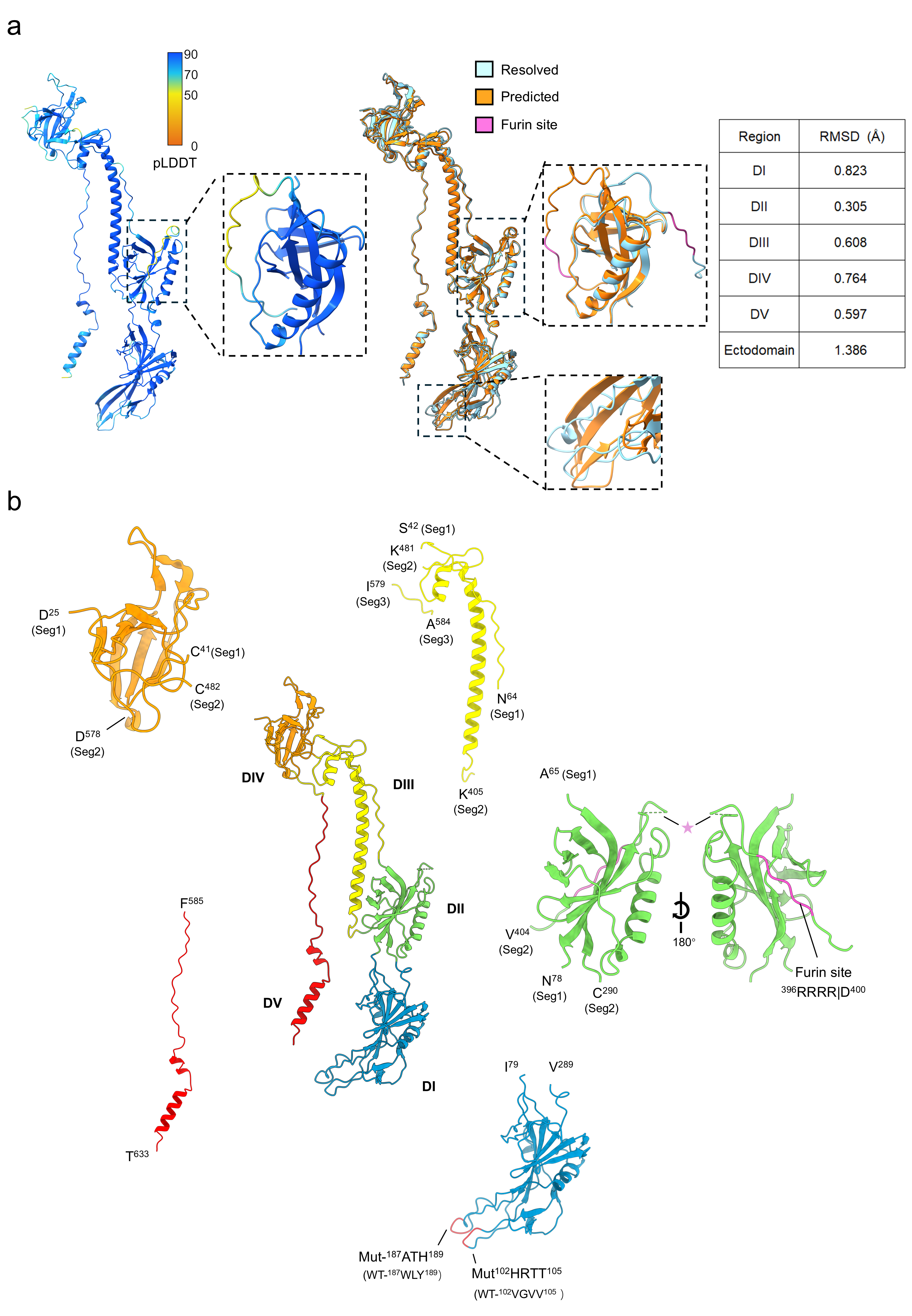

Supplement: S3 Fig — Zoomed-in views of selected regions highlight structural differences, including the DII–DIII linker containing the furin cleavage site and the fusion loop region. The table summarizes the RMSD values (in Å) between the predicted and resolved structures for individual domains and the ectodomain. b, The HHV-6B gB monomer is shown in cartoon form, with each domain colored as described in Fig 1C. The starting and ending residues of each domain segment are labeled. The fusion loops within DI are highlighted in red, with the mutated and wild-type amino acid sequences annotated for comparison. The furin cleavage site in the linker region between DII and DIII is colored pink, with the corresponding amino acid sequence and the precise furin cleavage site indicated. In the enlarged view of domain DII, the dashed segment marked by a pink asterisk indicates the unresolved region corresponding to residues 384–390 in the cryo-EM structure. (TIF) [file ppat.1013300.s003.tif]

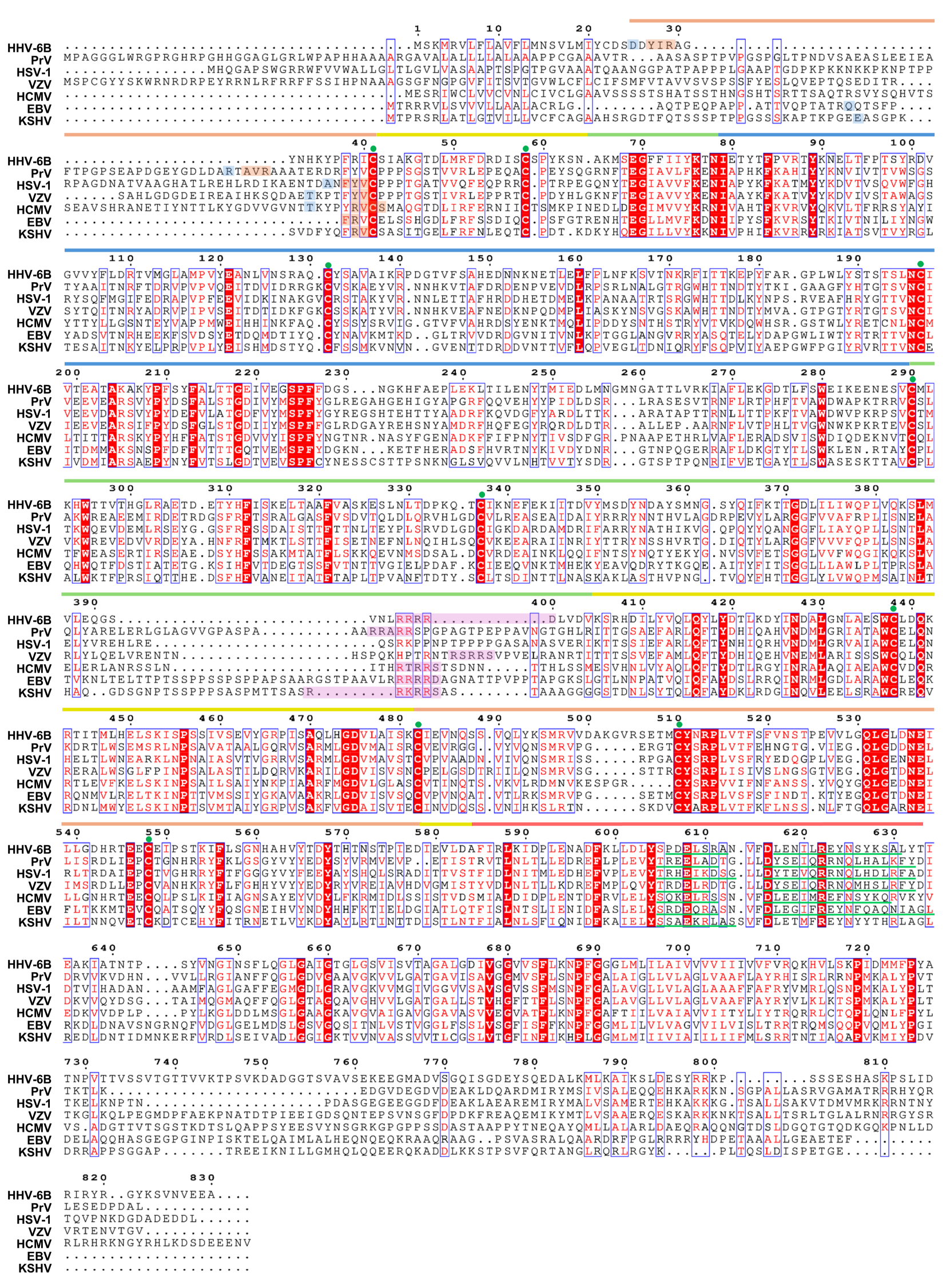

Supplement: S4 Fig — Multiple sequence alignment of HHV-6B gB with gB from HSV-1, VZV, HCMV, EBV, and KSHV was performed, including sequences of gB from HHV-6B (strain Z29), PrV (strain Kaplan), HSV-1 (strain KOS), VZV (strain Oka), HCMV (strain AD169), EBV (strain B95-8), and KSHV (strain GK18). Amino acids that are conserved across all seven gB are highlighted with red boxes and white text, while similar residues are shown in blue boxes with red text. The structural domains of HHV-6B gB are displayed above the alignment, colored following the scheme shown in Fig 1C. The first N-terminal residue resolved in the structures shown in Fig 2A is marked with a blue box, and the first N-terminal beta-strand is outlined with an orange box. Furin cleavage sites are marked with pink boxes. The two α-helices in the DV of each gB are marked with green lines beneath the sequences. The ten cysteine residues involved in disulfide bond formation are indicated with green dots above the aligned sequences. (TIF) [file ppat.1013300.s004.tif]

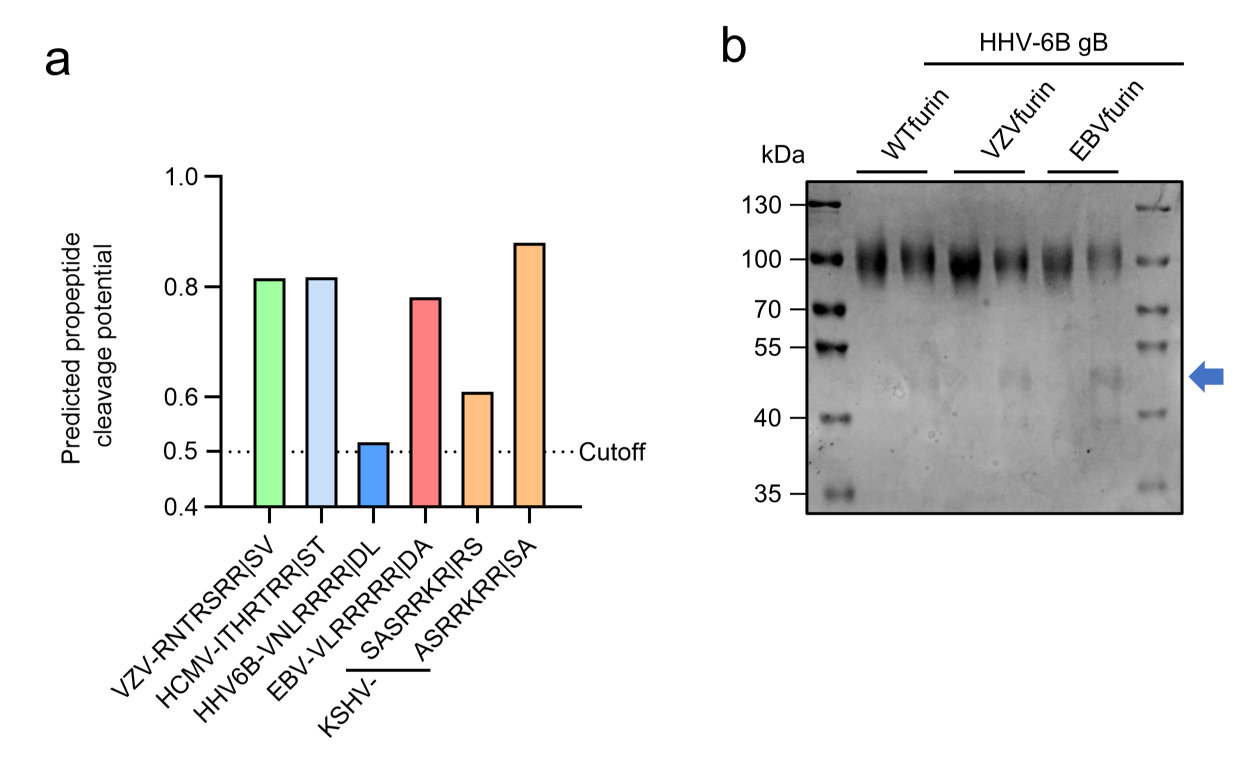

Supplement: S5 Fig — a, Furin cleavage potential was predicted via ProP-1.0, based on gB extracellular sequences of HHV-6B (strain Z29, accession P36320), HSV-1 (strain KOS, accession P06437), VZV (strain Oka, accession Q4JR05), HCMV (strain AD169, accession P06473), EBV (strain B95-8, accession P03188), and KSHV (strain GK18, accession F5HB81). The cutoff score, 0.5, indicates the presence of potential furin cleavage sites. b, SDS-PAGE analysis of purified HHV-6B gB ectodomains with wild-type or chimeric furin sites. The wild-type furin site (VNLRRRR|DL) was replaced with that of VZV (RNTRSRR|SV) or EBV (VLRRRRR|DA). All proteins were purified using the same method as the original HHV-6B gB680-mut construct. Blue arrow indicates the furin-cleaved fragment. (TIF) [file ppat.1013300.s005.tif]

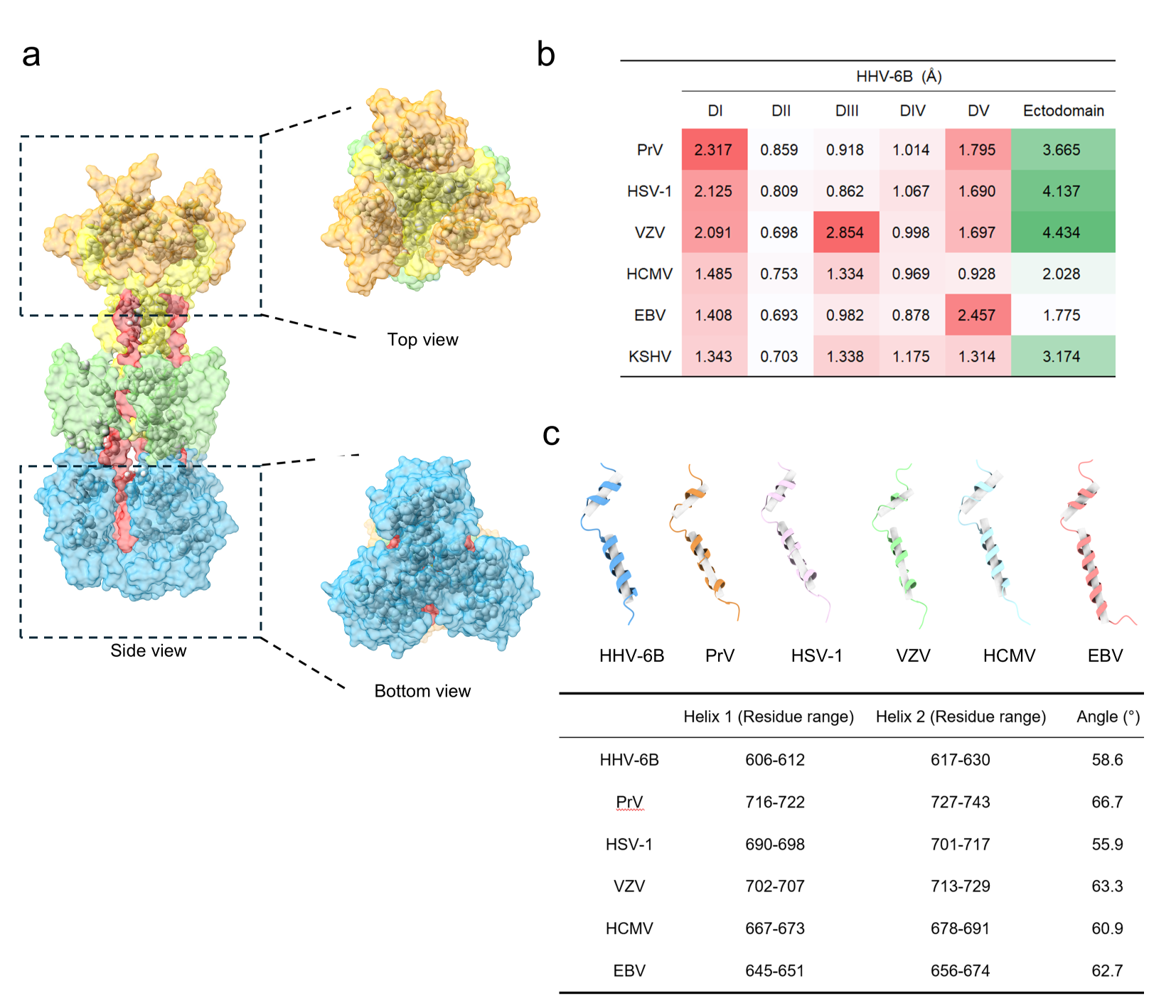

Supplement: S6 Fig — a, Conserved amino acids across HHV-6B, PrV, HSV-1, VZV, HCMV, EBV, and KSHV gB, as identified in S4 Fig, are mapped onto the resolved structure of HHV-6B gB. The HHV-6B gB is shown as a semi-transparent surface model, colored according to Fig 1C, with the conserved residues represented as opaque white spheres to illustrate the relative internal distribution of conserved amino acids within the gB structure. The side, top and bottom views are displayed. b, The resolved HHV-6B gB structure was aligned with gB from PrV (PDB: 6ESC), HSV-1 (PDB: 2GUM), VZV (PDB: 6VLK), HCMV (PDB: 5CXF), and EBV (PDB: 3FVC), and RMSD values (in Å) were calculated and displayed for each domain (DI–DV) and the ectodomain. For DI–DV, red shading indicates higher RMSD values and white indicates lower values. For the ectodomain column, green shading indicates lower RMSD values and white indicates higher values. c, Structural comparison of the two α-helices in DV from HHV-6B, PrV, HSV-1, VZV, HCMV, and EBV. The helices are depicted as cartoon, with the number of amino acids forming each helix and the angles between them displayed to illustrate structural differences. (TIF) [file ppat.1013300.s006.tif]

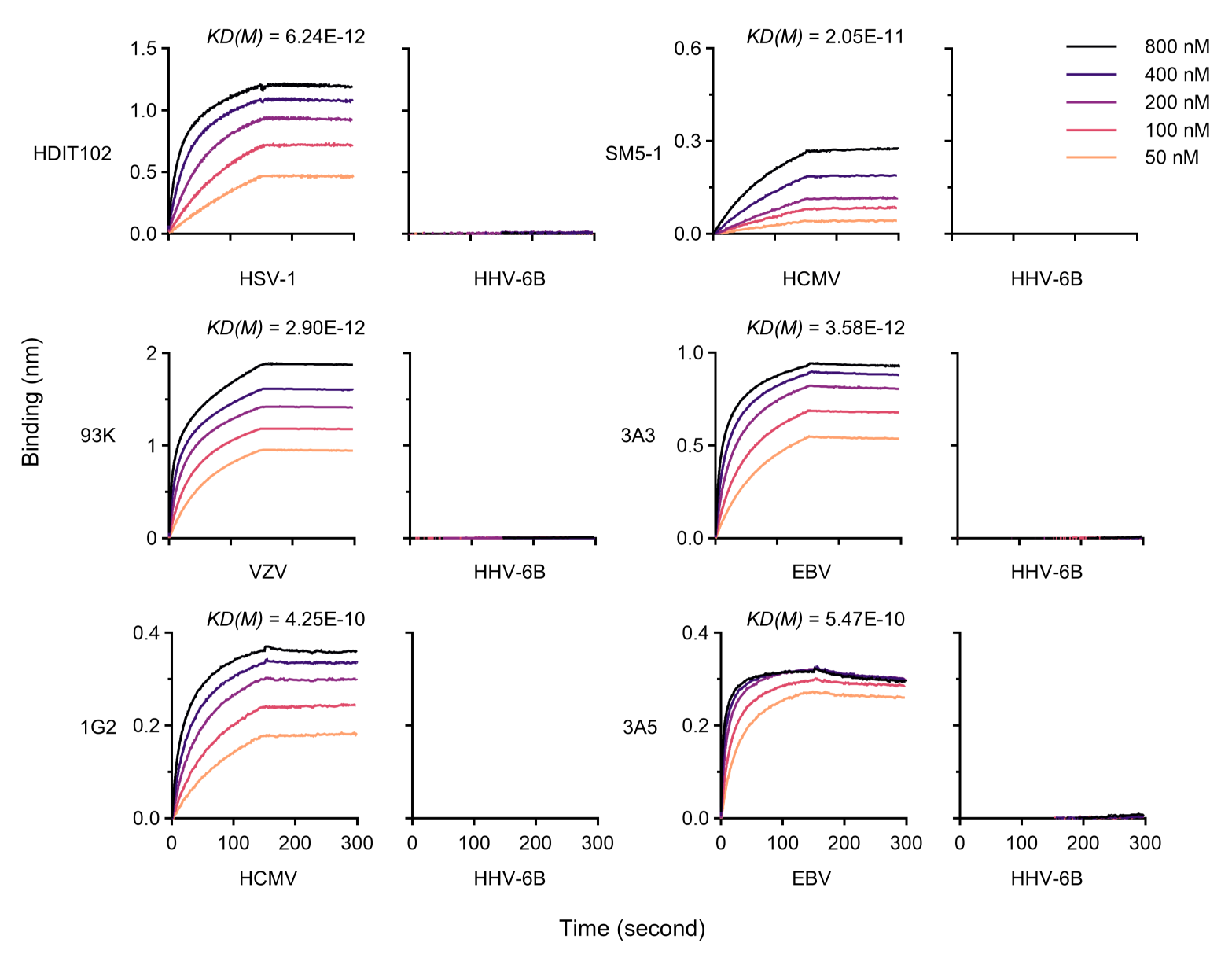

Supplement: S7 Fig — Biolayer interferometry (BLI) analysis was performed to evaluate the cross-reactivity of gB-specific neutralizing antibodies from various herpesviruses with HHV-6B gB. The antibodies tested included HDIT102 for HSV-1 gB, SM5–1 and 1G2 for HCMV gB, 93K for VZV gB, and 3A3 and 3A5 for EBV gB. Each antibody was tested for binding both to its respective target gB and to HHV-6B gB. The binding signal of 800, 400, 200, 100, and 50 nM of each gB associating with the corresponding ligand antibody captured on protein A biosensors is shown, followed by the dissociation phase. KD represents the equilibrium dissociation constant. (TIF) [file ppat.1013300.s007.tif]

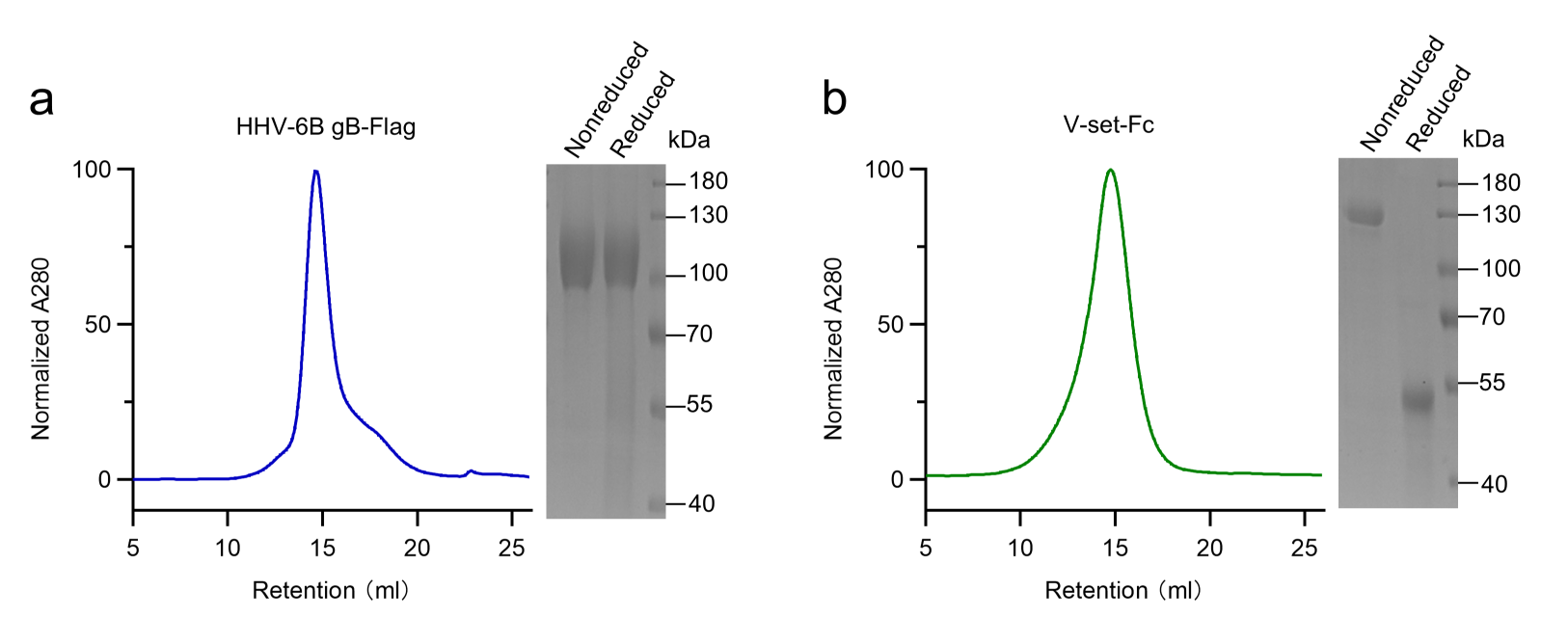

Supplement: S8 Fig — a, Size-exclusion chromatography (SEC) and Coomassie-stained SDS-PAGE analysis under reducing and non-reducing conditions of purified HHV-6B gB-Flag protein. b, Size-exclusion chromatography (SEC) and Coomassie-stained SDS-PAGE analysis under reducing and non-reducing conditions of purified V-set-Fc. (TIF) [file ppat.1013300.s008.tif]
