# Supplementary material for: Human herpesvirus 6B glycoprotein B postfusion structure, vulnerability mapping, and receptor recognition
Source: PLoS Pathog. 2025 Jul 9;21(7):e1013300. doi: 10.1371/journal.ppat.1013300 (PMC12240383; doi:10.1371/journal.ppat.1013300)
Supplement: S1 Table — (DOCX) [file ppat.1013300.s009.docx]

| **C3-symmetry HHV-6B (9JLI)** | |
| --- | --- |
| **Data collection and processing** | |
| Microscope | FEI Titan Krios |
| Camera | Gatan K3 |
| Magnification | 130,000 |
| Voltage(kV) | 300 |
| Electron exposure (e^–^/Å^2^) | 50 |
| Defocus range (μm) | -1.0 ~ -2.0 |
| Pixel size (Å) | 0.66 |
| Frames/movie | 32 |
| Movies (total) | 5,650 |
| Initial particle images (no.) | 1,677,197 |
| Final particle images (no.) | 272,190 |
| Symmetry imposed | C3 |
| **Map resolution (Å)** |  |
| FSC threshold | 0.143 |
| Map resolution range (Å) | 2 - 5 |
| **Model composition** |  |
| Non-hydrogen atoms | 14200 |
| Protein residues | 1806 |
| Ligands | 0 |
| **R.m.s. deviations** |  |
| **Bond lengths (Å)** | 0.004 |
| Bond angles (°) | 0.919 |
| **Validation** |  |
| MolProbity score | 1.78 |
| Clashscore | 7.92 |
| Rotamers outliers (%) | 0 |
| **Ramachandran plot** |  |
| Favored (%) | 95.02% |
| Allowed (%) | 4.98% |
| Disallowed (%) | 0 |
