# Supplementary material for: Human herpesvirus 6B glycoprotein B postfusion structure, vulnerability mapping, and receptor recognition
Source: PLoS Pathog. 2025 Jul 9;21(7):e1013300. doi: 10.1371/journal.ppat.1013300 (PMC12240383; doi:10.1371/journal.ppat.1013300)
Supplement: Attachment — Submitted filename: Response to Reviewers.docx [file ppat.1013300.s012.docx]

**Reply to the reviewers**

Reviewer #1:

**Part I - Summary**

In this paper the authors solve a cryo-EM structure of HHV-6B in the post fusion conformation.

Unsurprisingly it looks very similar to other herpesvirus fusion proteins. They can resolve the fuin cleavage site and it appears to be mostly uncleaved.

They authors also compare the structures of other gB proteins with Fabs bound to neutralizing epitopes but no Fabs bound to HHV-6B so this analysis doesn’t add much.

They provide biochemical data that gB binds to Nectin-2 via pulldown assays and model the nectin binding site into post-fusion gB and a hypothetical pre-fusion gB.

**Response**: Thank you for your comments and feedback on our study. Below, we address your concern and clarify aspects where needed.

**Part II – Major Issues: Key Experiments Required for Acceptance**

Introducing point mutations to confirm the nectin binding site on gB would be helpful in validating the model.

**Response**: Thank you for your suggestion. We would like to clarify that we have performed this experiment to validate the nectin-2 binding site on gB. As shown in Extended Data Fig. 8c, we introduced point mutations (Y132A, E150A, and N152A) at predicted interaction sites and assessed their impact on nectin-2 binding via immunoprecipitation (IP) assays (Response Fig.1). Our results show that these mutations partially reduced the interaction between gB and the nectin-2 V-set domain, supporting our structural model.

**Response Fig. 1|** For reference, Extended Data Fig. 8c shows the IP analysis of wild-type and mutant HHV-6B gB with V-set-Fc. The wild-type and mutant (ComA) gB proteins were incubated with V-set-Fc or an irrelevant human IgG control, followed by protein G pull-down and immunoblotting. The observed reduction in binding for the mutant supports the involvement of these residues in the gB-nectin-2 interaction.

Reviewer #2:

**Part I – Summary**

This paper reports the structure of HHV-6B glycoprotein B in the postfusion conformation at a resolution of 2.8 angstroms as determined by cryo-EM and single particle analysis. Despite the availability of many herpes glycoprotein structures, the fusion mechanism remains unclear. The findings reported in this paper are important and the manuscript is well-written. Please consider the following comments to improve the paper.

**Response**: Thank you for your careful review and positive assessment of our study. We value your constructive comments and have addressed them below.

**Part II – Major Issues: Key Experiments Required for Acceptance**

1.The structure of the HHV-6B gB cleavage site is of unclear significance. More context is needed for the reader. The functional significance of furin cleavage does vary among herpes gBs as the authors indicate (Line 679). However, many viral fusion proteins are functionally inactive in the absence of furin cleavage. It is important to note that that this is not the case for any of the herpesvirus gBs. Furin cleavage is not required for gB function. Indeed, the authors find that the HHV-6B cleavage site is poorly utilized. Please also emphasize that the function of furin cleavage of HHV-6B has not been evaluated.
**Response**: Thank you for your thoughtful suggestions. We acknowledge that while we have resolved the structure of the furin cleavage site in HHV-6B gB for the first time, the functional significance of furin cleavage in HHV-6B gB remains unclear. As you noted, providing more context on the role of furin cleavage in viral fusion proteins will help readers better interpret our findings.

In our initial discussion, we primarily highlighted studies on furin cleavage in other herpesvirus gBs, demonstrating that while furin processing can influence gB function, it is not strictly required for herpesvirus gB-mediated membrane fusion. We appreciate your suggestion to contrast this with other viral fusion proteins where furin cleavage is indispensable, as this provides an important comparative perspective and further highlights the unique properties of herpesvirus gB. In response, we have expanded our discussion accordingly.

Additionally, we have now explicitly stated that the functional role of furin cleavage in HHV-6B gB has not been evaluated, ensuring that our conclusions remain accurate and appropriately cautious. The revised discussion, with newly added or revised content highlighted, is shown below.

We appreciate your insightful feedback, which has improved the clarity and contextualization of our findings.

**Revised Discussion:**

In some viruses, such as flaviviruses and retroviruses, fusion protein activation is dependent on furin cleavage for functionality. In contrast, herpesvirus gB does not appear to strictly require furin cleavage for cell entry, and its functional significance varies across different herpesviruses. Furin cleavage can generate a CendR motif that is involved in binding to the b1 domain of NRP1, a reported receptor for EBV and KSHV gB. The furin cleavage motif in VZV gB contributes to pathogenesis in skin tissue in vivo. Besides, in PrV, the furin site in gB is essential for syncytium formation and pathogenesis *in vivo* but not for viral entry or replication. In this study, we provide structural insights into the HHV-6B gB furin site; however, its functional role in viral entry, infectivity, or pathogenesis remains to be elucidated. Our resolved structure of the furin site provides a structural foundation for future studies on the varying functions of the furin sites of HHV-6B and other herpesvirus gB.

2.Extended Fig 8c. The identification of the receptor-binding site on gB is not conclusive. Mutation of predicted gB-nectin-2 interaction sites decreases nectin-2 binding to an undetermined extent, but does not eliminate binding. This gB mutant is not characterized sufficiently. Do the three point mutations cause the protein to globally misfold, what is the effect of these mutations on cell surface expression, etc?
**Response**: Thank you for your insightful comments regarding the characterization of the gB mutant. We agree that assessing potential effects of the mutations on protein folding and membrane expression is important.

In our protein purification experiments, the size-exclusion chromatography (SEC) elution profile of the mutant (ComA) closely resembled that of HHV-6B gB-Flag, and the yield was robust, which could suggest that the introduced mutations do not result in substantial misfolding or aggregation during expression and purification (Response Fig. 2a).

To further evaluate cell surface expression, we constructed full-length HHV-6B gB (wildtype and ComA) with N-terminal or internal Flag tags (inserted after the signal peptide or within the DII–DIII linker near the furin cleavage site, respectively). These insertion sites were selected based on their flexible and solvent-exposed nature in the cryo-EM structure and prior studies showing their tolerance for tag insertion in HSV-1 and HCMV gB without disrupting function (Lin & Spear, 2007; Jean et al., 2000)^1,2^. We expressed both wild-type and ComA mutants in cells and performed anti-Flag immunostaining followed by confocal microscopy. As shown in Response Fig. 2b, although the staining intensity was modest, both wild-type and ComA constructs displayed similar plasma membrane localization regardless of tag position. These results indicate that the introduced mutations do not abolish trafficking to the membrane or prevent cell surface expression. The relatively weak surface staining is consistent with previous findings that HHV-6 gB exhibits limited membrane localization even in wild-type form, likely due to low steady-state surface levels or retention in intracellular compartments (Mahmoud et al., 2016)^3^.

Together, these results support that the observed reduction in nectin-2 binding is due to specific disruption of the binding interface, rather than a global misfolding effect. We hope our response addresses your concern.

**Response Fig.2| Characterization of wild-type and mutant HHV-6B gB**

**a**, Size-exclusion chromatography profiles of HHV-6B gB-Flag and HHV-6B gB ComA-Flag.

**b**, Full-length HHV-6B gB constructs carrying either an N-terminal Flag tag (N Flag) or an internal Flag tag inserted into the DII–DIII linker region (Linker Flag) were transfected into HEK293T cells. Both wild-type and ComA versions were included. At 36 h post-transfection, cells were fixed and stained with anti-Flag antibody under non-permeabilized conditions, followed by DAPI counterstaining. VC: vector control.

3.Fig. 4A is a confirmation of what has already been published in reference 14 and should be removed.
-Line 561: The AlphaFold2 modeling work presented does little to improve the understanding of structural details of gB-receptor interaction.
-Nectin-2 binding to prefusion gB is highly speculative. Experimental evidence is not provided. This interpretation should be limited to the Discussion and omitted from the Abstract.
**Response**:

We appreciate your concern and acknowledge that reference 14 (Ogawa et al., 2022)^4^ previously demonstrated the interaction between full-length HHV-6B gB and nectin-2 using co-transfection and immunoprecipitation (IP) in cells. Their truncation analysis further suggested that the V-set domain is responsible for binding. In our study, we sought to further validate and refine these findings by conducting IP with purified nectin-2 V-set and the gB ectodomain. This approach eliminates potential interference from intracellular components, allowing us to confirm the interaction under more controlled conditions. By using purified proteins, we provide additional support for the direct nature of this interaction, complementing the previous cellular studies. Given that our experiment builds upon and strengthens prior findings rather than simply replicating them, we believe that retaining Figure 4A adds value by providing a more refined mechanistic insight into gB-nectin-2 binding. We hope this clarification is helpful.

Regarding your comment on the AlphaFold2-predicted prefusion gB-nectin-2 interaction, we understand your concern that this model is speculative and does not provide conclusive evidence of receptor binding in this conformation. Without a stable prefusion HHV-6B gB, we are currently unable to directly validate its interaction with nectin-2 experimentally. Therefore, we present the AlphaFold2 model as a structural hypothesis rather than definitive evidence and in response to your suggestion, we have revised the Abstract to remove direct mention of prefusion gB-nectin-2 binding. Additionally, we have revised the Introduction and Discussion to explicitly acknowledge the speculative nature of this prediction. Specifically:

**Introduction:**

Original: "Furthermore, immunoprecipitation assay and structural modeling suggest that HHV-6B gB can bind nectin-2 in both prefusion and postfusion conformations, indicating an important role of nectin-2 in facilitating viral entry by interacting with multiple gB conformations."

Revised: "Furthermore, immunoprecipitation assay confirmed that nectin-2 interacts with the HHV-6B gB ectodomain, while structural modeling suggests that nectin-2 may also engage prefusion gB."

**Discussion:**

Original: "This suggests that nectin-2 potentially interacts with HHV-6B gB in both conformations, which may either facilitate viral attachment or assist in the process of membrane fusion."

Revised: "This suggests that nectin-2 potentially interacts with HHV-6B gB in both conformations, which may either facilitate viral attachment or assist in the process of membrane fusion, though this remains speculative and requires further validation."

We appreciate this valuable suggestion, which has improved the clarity and rigor of our manuscript.

4.The structure of pseudorabiesvirus gB has been reported PMID: 29046441. This structure should be considered in the analyses presented.

**Response**: Thank you for this valuable suggestion. While our initial analysis focused on human herpesvirus gB structures, we fully agree that pseudorabies virus (PrV), as a representative α-herpesvirus with a high-resolution gB structure (PDB: 6ESC; PMID: 29046441), offers important comparative insights.

Following your suggestion, we have incorporated the PrV gB structure into our analyses, making modifications to Figure 2d, 2e, Extended Data Fig. 4, 6a, 6b, their legends and updating the corresponding sections in the Results (Response Fig. 3). This inclusion has helped provide a broader perspective on gB structural conservation and variability across herpesviruses.

We appreciate your recommendation, which has strengthened our manuscript.

**Response Fig. 3| Updated structural comparison including PrV gB**

Panels correspond to revised versions of Fig. 2d, Fig. 2e, Extended Data Fig. 4, 6a, and 6b in the manuscript. The PrV gB sequence (UniProtKB accession number: G3G8X1) or structure (PDB: 6ESC) was included as an additional α-herpesvirus reference to enhance cross-species comparisons of gB sequential or structural conservation and domain-specific RMSD values.

**Part III – Minor Issues: Editorial and Data Presentation Modifications**
Reviewer #2: 1. The title should include a postfusion descriptor.

**Response**: We agree that explicitly indicating the postfusion conformation of the resolved HHV-6B gB structure enhances clarity and better aligns with the study's focus. In response, we have revised the title to: "Human herpesvirus 6B glycoprotein B postfusion structure, vulnerability mapping, and receptor recognition." We appreciate this recommendation, which has improved the precision of our manuscript's presentation.

Reviewer #3:

**Part I – Summary**

In this paper, Xie et al. provide an intriguing set of data detailing the structural differences between postfusion gB of HH6B and the gB structures of other Herpesviruses. The study successfully resolved the cryo-EM structure of the HHV-6B gB ectodomain, revealing several unique features, including a well-structured N-terminal region starting from residue 25. In addition, the Furin cleavage site was structurally resolved, providing a first structural description of this functional region found in many gB proteins. The authors also confirmed the interaction between HHV-6B gB and the receptor nectin-2, suggesting this binding could facilitate viral attachment or fusion. Moreover, conserved binding sites on gB were identified that may serve as targets for neutralization and offer insights for the development of targeted therapeutic strategies.
 Overall, the study is technically well conducted, although there is almost no functional data that would corroborate the claims and assumptions derived from the determined structure. Therefore, there are several points the authors should address, before the paper can be considered for publication.

**Response**: Thank you for your thoughtful review and for recognizing the structural insights of our study. Below, we address your specific concerns in detail.

**Part II – Major Issues: Key Experiments Required for Acceptance**

1) The new postfusion structure for HHV6B gB presented here shows a very high resemblance with previously determined structures of other herpesviruses. The authors should prepare an alphafold model of HHV6B and see how it compares with the determined structure. Are there structural features that alphafold was not able to predict that would be functionally or immunologically interesting?

**Response**: Thank you for this insightful suggestion. In response, we have generated an AlphaFold3 model of HHV-6B gB ectodomain and performed a structural comparison with our experimentally determined postfusion structure (Response Fig. 4).

Overall, the predicted structure aligns well with our cryo-EM model. The AlphaFold3 model recapitulated key structural features, including the outer positioning of the N-terminal β-strand in DIV. However, several structural differences were observed. For example, angular deviations between structural elements were observed. Moreover, we noted significant divergence in the linker connecting DII to the DIII base, which harbors the furin cleavage site. In this region, the AlphaFold3 model showed a markedly low pLDDT score, suggesting that AlphaFold3 may not reliably capture the structural properties of this flexible and functionally relevant region.

Additionally, the fusion loops—which are critical for membrane fusion—also showed differences. While our construct includes (mutations in the fusion loops to reduce aggregation (a common strategy in herpesvirus gB structural studies), previous work has shown that such substitutions have minimal impact on overall structure (Oliver et al., 2021)^5^. In our cryo-EM structure, the DI fusion loop adopts an inward and upward orientation, whereas in the AlphaFold3 model, it projects downward, highlighting another region where modeling may not fully recapitulate conformational preferences observed in experimentally determined structures.

We have included the AlphaFold3-predicted model in Extended Data Fig. 3a for readers’ reference. Your suggestion has helped to strengthen our study and provided further structural insights into HHV-6B gB.

**Response Fig. 4| Structural comparison between AlphaFold3-predicted HHV-6B gB and the cryo-EM model.**

Left: AlphaFold3-predicted structure colored by pLDDT confidence scores. Right: Structural alignment of the predicted (orange) and resolved (light blue) structures, with key differences highlighted in the N-terminal β-strand, the DII–DIII linker (furin site), and the DI fusion loop region.

2) In line 241 the authors write: ‘The unique sequence and structural features of the N-terminus of HHV-6B gB may contribute to its distinct functional and immunogenic properties.’ And also, in the discussion that ‘…its unique short N-terminus and the distinct position of its first b-strand may lead to functional and immunogenic differences.’. The position and sequence difference may well account for immunogenic differences, but how this arrangement could contribute a functional difference compared to other gBs should be better described. It would be good if the authors could elaborate more on this, also putting this in context with the recently published pre-print (https://doi.org/10.1101/2024.10.23.619923). For example, would the N-term generally have a prefusion stabilising role that is missing in HHV6B?

**Response**: Thank you for this insightful suggestion. Although still in the preprint stage, the study you referenced provides highly significant structural insights into prefusion-stabilized herpesvirus gB trimers. This work represents a major step forward in addressing the long-standing challenge of stabilizing and obtaining prefusion gB structures. Your suggestion is particularly constructive, as this study reveals N-terminal structural features absent in previously determined postfusion gB structures, offering relevant insights for our work. Specifically, the VZV gB N-terminus was found to interact with the DI hinge in the prefusion state, suggesting a potential role in stabilizing this conformation and regulating fusion activation. In contrast, HHV-6B gB has a shorter N-terminal region, which may imply functional differences in how its DI hinge is stabilized. However, as PDB coordinates for the prefusion structures are not yet available, a more detailed comparison remains limited. Nonetheless, your suggestion has helped refine our interpretation of the gB N-terminal function, and we have expanded our discussion accordingly, as detailed below.

**Expanded Discussion:**

Interestingly, a recent study described the prefusion-stabilized structures of multiple herpesvirus gBs, in which portions of the N-terminal regions were observed (McCallum & Veesler, 2024)^6^. These findings revealed that gB N-terminus adopt two main interaction patterns: binding either to DIV or to the DI hinge. Among them, the N-terminus of VZV gB was resolved in the most detail, showing clear interactions with the DI hinge, which suggests a potential role in stabilizing the prefusion state and regulating fusion activation. However, this structural feature was not observed in EBV gB, indicating that not all herpesvirus gBs utilize their N-terminal regions in the same way.

3) It would be great to understand the functional role of the furin cleavage site better. One could e.g. replace it with the VZV cleavage site to make it more efficient and compare it to a mutated version that is not cleavable anymore. But I understand that functional studies for this i.e. in a virus background or in a cell-cell fusion assay is not trivial, but could be done, as shown in Ref. 29.

**Response**: Thank you for this valuable suggestion. We agree that understanding the functional role of the furin cleavage site through mutagenesis and cell–cell fusion assays would significantly enhance the mechanistic depth of our study.

In response, we first evaluated the effect of furin site sequence on cleavage efficiency. Based on the HHV-6B gB680-mut construct, we generated additional gB ectodomain variants in which the native furin site was replaced with the corresponding sequence from EBV or VZV, purified them, and assessed their processing using SDS-PAGE analysis. Both chimeric constructs showed increased levels of cleaved protein compared to the wild-type furin site, suggesting that the low furin cleavage efficiency of HHV-6B gB is at least partly attributable to its native cleavage motif (Response Fig. 5a). Then, we attempted to evaluate the impact of furin cleavage by generating a series of constructs, including full-length HHV-6B gB, a cleavage-deficient mutant in which the furin site was replaced with a GGGGS linker, and chimeric constructs in which the HHV-6B furin site was substituted with that of EBV or VZV, which all contained the Y822A mutation that was previously reported to enhance membrane localization and fusion efficiency (Wakata et al., 2018; Wakata et al., 2021) ^7,8^. In parallel, we prepared full-length HHV-6B gH, gL, gQ1, gQ2, and CD134 constructs, as well as luciferase and T7 plasmids for use in a standard cell-cell fusion assay. We evaluated fusion under different transfection conditions and across 293T-293T and 293T–CHO fusion pairings but despite extensive efforts, we were unable to detect a robust fusion signal using wild-type or furin-site–modified HHV-6B gB. We have also tried wild-type HHV-6B gB without the Y822A mutation, and combined overexpression of CD134 and nectin-2, but the fusion activity remained weak and variable. Interestingly, during the course of troubleshooting, we observed a clear fusion signal when co-expressing HHV-6B gH/gL/gQ1/gQ2 and T7 polymerase with HCMV gB (in place of HHV-6B gB), and overlaying with cells expressing CD134 and luciferase. This result, reminiscent of previous KSHV fusion studies where EBV gB could substitute for the inefficiently fusogenic KSHV gB, supports the functional integrity of the HHV-6B gH/gL/gQ1/gQ2–CD134 axis and suggests that HHV-6B gB may require further optimization for robust fusion readouts in heterologous expression systems. Although we were ultimately unable to establish a reliable HHV-6B gB-based fusion assay, we have made substantial efforts in this direction and appreciate your recommendation, and hope our efforts demonstrate our commitment to addressing this question.

**Response Fig. 5| Assessment of furin site variants of HHV-6B gB.**

**a**, SDS-PAGE analysis of purified HHV-6B gB ectodomains with wild-type or chimeric furin sites. The wild-type furin site (VNLRRRR|DL) was replaced with that of VZV (RNTRSRR|SV) or EBV (VLRRRRR|DA). All proteins were purified using the same method as the original HHV-6B gB680-mut construct. Blue arrow indicates the uncleaved gB ectodomain; green arrow indicates the furin-cleaved fragment.

**b**, Effector cells were co-transfected with full-length HHV-6B gH/gL/gQ1/gQ2 and T7 polymerase, along with either empty vector (VC), full-length HHV-6B gB containing a Y822A substitution, or HCMV gB. Target cells were transfected with a T7-driven luciferase reporter plasmid, with or without CD134.

4) For the nAB epitope comparison the authors map the interacting residues of several nABs on HHV6B. Judging from the EM density shown in Fig. 1, the glycosylations on gB should be visible. There are significant differences in the number of glycosylations on gB from different species, which might cover immunologically important epitopes and this should be considered here.

**Response**: Thank you for this important suggestion. We agree that glycosylation is an important factor to consider when evaluating neutralizing antibody (nAb) epitope accessibility, as glycan shielding can influence immunogenicity. In our revised analysis, we examined the glycosylation sites resolved in the HHV-6B gB cryo-EM structure, as well as those observed in the structures of other nAb-gB complex, and included these features when mapping aligned nAb footprints (Response Fig. 6).

Specifically, in the HHV-6B gB structure, we resolved two N-linked glycans, one of which corresponds to N^247^ and is aligned with D^323^ in HSV-1 gB, an interacting residue for the nAb HDIT102. While the lack of sequence conservation at this site likely underlies the observed lack of cross-reactivity, the presence of an N-linked glycan at this position in HHV-6B gB may additionally reduce the accessibility of this epitope region. Additionally, we noted that in other herpesvirus gB structures, several nAb footprints are also located near or overlap with glycosylated residues. Notably, one of the SM5-1 contact residues in HCMV gB, N^383^, is glycosylated, and the aligned position in HHV-6B gB, N^329^, is also glycosylated. This suggests that antibody binding and glycosylation are not necessarily mutually exclusive, and their compatibility may depend on glycan orientation and flexibility.

We have updated the figure to highlight these resolved glycosylation sites and incorporated relevant observations into the Result section to address their potential impact on nAb binding and epitope accessibility. This valuable point has helped to improve the immunological interpretation of our structural comparisons.

**Response Fig. 6| Glycosylation considerations in neutralizing antibody (nAb) epitope mapping.**

Aligned neutralizing antibody footprints from HSV-1, VZV, HCMV, and EBV gB structures were mapped onto corresponding HHV-6B gB domains. Key interacting residues are labeled, with conserved residues between HHV-6B and other gBs highlighted. Resolved glycans within the displayed regions are shown as sticks. Glycosylated residues identified in the original antibody–gB complex structures are shown in bright yellow, while those resolved in the HHV-6B gB cryo-EM structure are shown in hot pink, in both surface and cartoon representations.

5) The authors have set up a nice BLI assay system to check for interactions of different gBs with nABs. It would be good to report the different affinities of the different nABs. If possible, it would also be good to use the same assay system to determine the binding affinity of nectin-2 to gB and also the interaction mutant ComA.

**Response**: We have performed BLI assays with nAbs binding to serial dilutions of different gB, and have globally fitted the affinity values, which are now shown in the updated Extended Data Fig. 7a (Response Fig. 7).

Regarding your suggestion to determine the binding affinity of nectin-2 to HHV-6B gB or its mutant, we fully agree that this would provide valuable insights. We attempted these measurements; however, the binding response in our BLI assays was weak and did not yield a reliable affinity measurement. This discrepancy between IP-based detection and BLI measurements is not unexpected. In IP experiments, protein interactions occur in solution over an extended incubation period, which can enhance even weak interactions. Additionally, Western blot detection further amplifies signal intensity, increasing the likelihood of detecting low-affinity binding. In contrast, BLI requires a more stable interaction with a detectable response shift, and weak interactions may fall below the detection threshold, particularly when the dissociation rate is high, which is commonly observed in herpesvirus gB–receptor interactions (Zhong et al., 2023)^9^. Furthermore, AlphaFold predictions suggest that the HHV-6B gB-V-set interface involves a limited number of amino acid interactions, which may suggest that the interaction between gB and nectin-2 is relatively weak and potentially transient, and aligns with our BLI results.

We appreciate your insightful suggestion, and we hope that our explanation addresses your concern.

**Response Fig. 7| BLI analysis of cross-reactivity and binding affinities of neutralizing antibodies against herpesvirus gBs.**

Binding kinetics of six neutralizing antibodies—HDIT102 (HSV-1), SM5-1 and 1G2 (HCMV), 93K (VZV), and 3A3 and 3A5 (EBV)—to their respective gB antigens and to HHV-6B gB were measured by biolayer interferometry (BLI). Serial dilutions of gB proteins (800, 400, 200, 100, and 50 nM) were tested, and the corresponding association and dissociation phases are shown. KD values were globally fitted using a 1:1 binding model. No appreciable binding to HHV-6B gB was detected for any of the non-HHV-6B nAbs, supporting the lack of cross-reactivity observed in Fig. 3.

6) Did the authors try to determine a structure of the gB-nectin2 complex with cryoEM? This would be a great way to verify the prediction.

**Response:** Thank you for this thoughtful suggestion. As mentioned above, the interaction between HHV-6B gB and the nectin-2 V-set domain is relatively weak, preventing us from obtaining a stable complex for cryo-EM analysis. Given these limitations, alternative approaches such as X-ray crystallography, crosslinking, or antibody-assisted stabilization may be needed to resolve the interaction in future structural studies.

**Part III – Minor Issues: Editorial and Data Presentation Modifications**
1) The authors show that the ectodomain of HHV6B gB can be purified using a fusion loop mutant in contrast to the wt protein construct. In ExtData Fig. 1b the two preparations are shown in SDS PAGE. It would be interesting to what degree the protein is cleaved by furin which could done using reducing conditions. There is no loading control, so it is really hard to tell what the samples really are and how much of each was loaded. It might be more informative to also see the expression levels of these constructs e.g. in cell lysates.

**Response:** Thank you for this insightful comment. To evaluate furin cleavage in our purified HHV-6B gB ectodomain preparations, we compared the cleavage status under both reducing and non-reducing conditions. As shown in Extended Data Fig. 5a (Response Fig. 8a), equal amounts of A280-quantified purified proteins were loaded per lane, and Coomassie Brilliant Blue staining was used to visualize total protein. The similar band intensities observed between lanes indicate comparable loading. Regarding Extended Data Fig. 1b, the wild-type and mutant gB samples were subjected to identical purification and concentration protocols, and equal volumes were loaded for SDS-PAGE. The absence of a detectable band in the wild-type lane likely reflects its poor recovery during purification, rather than differences in loading. Besides, we previously evaluated the expression of wild-type and mutant gB constructs in cell supernatants by Western blot (Response Fig. 8b), using equal plasmid input and equal supernatant volumes for loading. Consistent with the purification results, the wild-type gB construct showed minimal signal, while fusion loop mutants yielded clearly detectable expression. This is in agreement with prior observations and may be due to reduced secretion or aggregation of wild-type gB, especially in PBS-based purification systems. We hope our clarification addresses your concern.

**Response Fig. 8| Assessment of HHV-6B gB purification and expression**

**a,** SDS-PAGE analysis under reducing and non-reducing conditions of purified HHV-6B gB ectodomain (680-mut). Equal amounts of A280-quantified protein were loaded per lane. Coomassie Brilliant Blue staining was used to visualize total protein, and comparable band intensities indicate equivalent loading.
**b,** Western blot detection of HHV-6B gB ectodomain expression from various truncation constructs, including wild-type and fusion loop–mutated versions. Plasmids encoding His-tagged gB constructs were transiently transfected into 293T cells, and supernatants collected at 36 h post-transfection were loaded in equal volumes. Anti-His antibody was used for detection. The two wild-type constructs (wt-trunc.1 and wt-trunc.2) show minimal expression compared to their fusion loop–mutated counterparts.

2) In HSV-1 gB, DII features an alpha helix (helix X) that was only resolved in a later crystal structure by Cooper et al. (10.1038/s41594-018-0060-6). Is this helix also present in HHV6B and if yes, was it resolved in the structure?

**Response:** Thank you for this thoughtful question. We would like to clarify that the α-helix referred to as “helix X” in HSV-1 gB DII was first resolved in the crystal structure reported by Stampfer et al. (*J Virol*, 2010; PDB: 3NWA)^10^. In that study, uncleaved HSV-1 gB ectodomains were crystallized, allowing visualization of an α-helix formed by residues 462–477 that was absent in the earlier structure. To evaluate whether a similar helix is present in HHV-6B gB, we aligned the DII region of our cryo-EM structure with HSV-1 gB DII (PDB: 3NWA). The corresponding segment in HHV-6B adopts an extended loop conformation and does not form a resolved α-helix (Response Fig. 9a). We further examined the cryo-EM structure of VZV gB (PDB: 6VLK), an α-herpesvirus gB, and found that this region was unresolved, suggesting structural flexibility of this region in VZV gB DII (Response Fig. 9a)^5^. Moreover, multiple sequence alignment revealed limited conservation across herpesvirus gBs in this segment, consistent with the observed structural variation and indicating that this region may exhibit virus-specific conformational properties (Response Fig. 9b). We hope this addresses your question.

**Response Fig. 9| Structural and sequence comparison of the helix X region in DII across herpesvirus gBs.**

**a**, Structural alignment of HSV-1 gB DII (PDB: 3NWA, green), HHV-6B gB DII (PDB: 9JLI, cyan), and VZV gB DII (PDB: 6VLK, red) highlights the region corresponding to helix X (HSV-1 residues 462–477). In HSV-1 gB, this segment forms an α-helix (helix X), In VZV gB, this region is not resolved in the cryo-EM density map, indicated by a dashed red line.

**b**, Multiple sequence alignment of the helix X region and its flanking residues across herpesvirus gBs. The green line above the alignment indicates the region corresponding to HSV-1 helix X (residues 462–477).

3) Fig. 1 c) It would be nice to indicate the regions that were not modelled in the structure. Fig. 1 d) Please mark the N- and C-terminus with the first/last built residue number as well as where loops are missing, with residue numbers.

**Response:** Thank you for this helpful suggestion. In response, we have updated Fig. 1c to more clearly indicate the regions that were not modeled in the structure (Response Fig. 10a). Regarding Fig. 1d, we would like to note that the first and last built residue numbers for each domain were previously provided in Extended Data Fig. 3, where the domains are shown in enlarged views for clarity. Nevertheless, we agree that labeling the termini directly in Fig. 1d would improve clarity. Therefore, we have now added annotations for the N- and C-termini of the resolved structure in Fig. 1d (Response Fig. 10b). As for the missing loop region, which corresponds to a eight-residue linker between domains DII and DIII, we found that marking it directly in Fig. 1d would be visually unclear. To address this, we have now added a specific annotation for this missing loop Extended Data Fig. 3 (Response Fig. 10c), along with a brief explanation in the legend.

We appreciate this suggestion, which has helped improve the clarity of our structural representation.

**Response Fig. 10| Structural annotation updates addressing missing regions and termini in HHV-6B gB.**

**a**, Full-length sequence schematic of HHV-6B gB with domain organization and structural features. Regions not resolved in the cryo-EM map are marked with red bars.

**b**, Trimeric cryo-EM structure of HHV-6B gB with N-terminal (D²⁵) and C-terminal (T⁶³³) residues annotated.

**c**, Enlarged view of domain DII from Extended Data Fig. 3b, with the unr	esolved loop (residues 384–390) between DII and DIII indicated by a dashed segment and pink asterisk.

4) Fig. 2 – the arrangement of the individual panels is a bit awkward, especially panel d. This should be rearranged in order to group things that go together.

**Response:** Thank you for your suggestion. We have rearranged Figure 2, especially panel d, to improve clarity and better group related elements together (Response Fig. 11).

**Response Fig. 11| Revised layout of Fig. 2d to improve clarity and grouping.**

Panel d from Fig. 2 has been rearranged to more clearly associate the sequence conservation map, domain segmentation bar, and corresponding structural representation.

5) Concerning the furin cleavability – the position of the cleavage site on DII and the possibility to resolve it might mean that the site is not fully accessible to the enzyme, as it tightly interacts with the surface of DII. The question is if this would be the same situation in prefusion conformation. The ectodomain construct most probably adopts the postfusion conformation almost instantly after translation due to the missing transmembrane parts and before the posttranslational modifications are done. But in context of the full-length protein, the situation might be different. The authors should comment on this.

**Response:** Thank you for raising this important point. We agree with your interpretation that the gB ectodomain construct likely adopts the postfusion conformation rapidly upon secretion due to the absence of the transmembrane region, prior to completion of post-translational modifications. This experimental context differs from the native full-length protein embedded in the viral membrane, which may allow a more flexible prefusion conformation and potentially different exposure of the furin cleavage site. Although our study included comparative analyses with other herpesvirus gB ectodomains and in vitro furin cleavage assays to control for some of these variables, we cannot rule out the possibility that furin processing may differ between postfusion ectodomains and full-length prefusion gB. We have added the following clarification to the Discussion to address this important point (see below). We appreciate your thoughtful comment, which helped us better frame the limitations and biological implications of our cleavage analysis.

**Revised Discussion:**

Interestingly, we found that the proportion of cleaved gB in our purified protein was low, and an in vitro assay confirmed that the ectodomain of HHV-6B gB is highly resistant to furin cleavage, potentially due to the sequence preferences of furin. Notably, the recombinant HHV-6B gB ectodomain analyzed in our study is expected to rapidly adopt a postfusion conformation upon translation given the absence of the membrane anchor, and whether furin cleavage occurs similarly in full-length, prefusion gB remains to be determined.

6) In ExtData Fig. 5b, for KSHV there seem to be two possible recognition sites, with one having a higher cleavage potential than the other. Does the analysis done in c) then suggest, that the less efficient cleavage site is the one accessible for the enzyme?

**Response:** Thank you for your insightful question. In our analysis, we used ProP-1.0 with the furin-specific prediction mode, which evaluates cleavage potential based on primary sequence motifs (Duckert, 2004). For KSHV gB, this analysis identified two potential furin cleavage sites, with one site having a higher predicted cleavage potential. However, our in vitro cleavage assay showed that KSHV gB was processed with relatively low efficiency, raising the question of whether the experimentally cleaved site corresponds to the one with the lower predicted score. This is possible, as cleavage efficiency is influenced not only by sequence motifs but also by structural factors such as local folding, which may affect site accessibility. While the two predicted sites differ by only a single amino acid and this region was not resolved structurally, their actual accessibility remains uncertain. Additionally, while ProP-1.0 is highly sensitive, as with any predictive model, occasional discrepancies between predicted and actual cleavage sites can occur. This is also why we conducted in vitro furin cleavage assays to experimentally assess cleavage efficiency across different gB proteins. We appreciate your comment and hope this clarification is helpful.

7) In ExtData Fig. 5c it might be better to mark the bands with an asterisk or point next to the band, instead of above, to make the position clearer. I don’t understand the difference between ‘uncleaved portion’ and ‘faint uncleaved extracellular portion’. If one compares VZV and EBV, the ratio of cleaved and uncleaved seems similar (as far as I can tell).

**Response:** We agree that marking the bands with an asterisk next to them would improve clarity and we have made this adjustment in Extended Data Fig. 5c accordingly. As shown below (Response Fig. 12) we have added a light blue asterisk in the upper-left corner of the uncleaved bands to more clearly indicate their positions. Regarding the distinction between the 'uncleaved portion' and the 'faint uncleaved extracellular portion,' we acknowledge that this terminology may have caused confusion. To improve clarity, we have removed these separate labels and now describe both as largely cleaved in figure legend. We appreciate this valuable feedback, which has helped improve the clarity and presentation of our data.

**Response Fig. 12|** SDS-PAGE analysis of purified, reduced ectodomains (ECD) of gB from HSV-1, VZV, HCMV, HHV-6B, EBV, and KSHV, without furin site mutations. Light blue asterisks mark uncleaved gB bands, placed next to the corresponding lanes for clarity, as suggested. This updated figure corresponds to Extended Data Fig. 5c in the revised manuscript.

8) From line 459 the authors write ‘Although the overall structures of the analyzed domains of different herpesvirus gB and HHV-6B gB are highly similar, especially for DII and DIV, …’ while before they claim that (line 421) ‘variations in DI, DII, and DIV account for the majority of interspecies and intraspecies differences.’ One of the two claims should be revised to keep it consistent.

**Response:** Thank you for your careful attention to detail and we understand the potential confusion regarding these statements. In the statement on line 421, our intent was to describe sequence-level variability between HHV-6A and HHV-6B gB, highlighting that DI, DII, and DIV harbor most of the sequence differences across species and strains. In contrast, the later statement on line 459 refers specifically to structural conservation. To avoid confusion, we have revised the corresponding sentence, as shown below. We appreciate your valuable feedback in refining our manuscript.

**Original (Line 419-422):** Our analysis of gB sequence conservation between HHV-6A and HHV-6B, coupled with the resolved HHV-6B gB structure, highlights the marked conservation of DIII and DV, whereas variations in DI, DII, and DIV account for the majority of interspecies and intraspecies differences.

**Revised:** Our analysis of sequence conservation between HHV-6A and HHV-6B gB, mapped onto the resolved HHV-6B gB structure, indicates that DIII and DV are highly conserved at the sequence level, whereas DI, DII, and DIV exhibit more sequence divergence, accounting for most of the interspecies and intraspecies variability.

9) ExtData Fig. 6 – it would be informative to have an overview of the RMSDs between the individual domains. Also, please provide the RMSD values with a unit (Å?).

**Response:** Thank you for this helpful suggestion. We have now updated the Extended Data Fig. 6b and added an overview of the RMSD values between individual domains of HHV-6B gB and those of other herpesvirus gBs (Response Fig. 13) and the appropriate unit (Å) has also been included for clarity. In addition, we have carefully reviewed the Results section and added the appropriate unit (Å) where RMSD values were previously mentioned without a unit.

**Response Fig. 13|** RMSD values (in Å) between HHV-6B gB and other herpesvirus gBs calculated by domain (DI–DV) and ectodomain. For DI–DV, red shading indicates higher RMSD values and white indicates lower values. For the ectodomain column, green shading indicates lower RMSD values and white indicates higher values.

10) Fig. 3 - Although colour coded, it would be helpful to put the names of the nABs to the corresponding structures in panel a.

**Response:** Thank you for your suggestion. We have now added the names of the nAbs to the corresponding structures in Fig. 3a for better clarity (Response Fig. 14).

**Response Fig. 14|** Structural visualization of herpesvirus-neutralizing antibodies (nAbs) bound to their respective gB proteins. Each antibody is labeled with its name and shown in cartoon representation.

11) Line 588: is the number in parenthesis (m = 1.720) the calculated RMSD?

**Response:** Thank you for your careful review. We have now corrected the RMSD value notation in line 588 accordingly.

12) The interaction site of Nectin-2 with DI is very close to the membrane. Would the receptor be able to reach that far and so close to the viral membrane?

**Response:** Thank you for your thoughtful question. We acknowledge that the predicted nectin-2 interaction site on HHV-6B gB DI is positioned relatively close to the viral membrane. However, we believe this does not necessarily preclude receptor engagement for several reasons. First, both HHV-6B gB and nectin-2 are likely to extend further from the membrane than shown in the model. The extracellular portion of HHV-6B gB in our study does not include the membrane-proximal region (MPR), which has been observed in the full-length postfusion structure of HSV-1 gB to separate DI from the membrane (Cooper et al., 2018)^11^. Similarly, while our study uses only the nectin-2 V-set domain (residues 1-158), full-length nectin-2 consists of 538 residues, including additional extracellular Ig-like domains, which likely provide further spatial extension from the membrane. Additionally, our AlphaFold-predicted model suggests that the interaction between HHV-6B gB and nectin-2 may not be restricted to the postfusion conformation. Given the dynamic nature of gB transitions, it remains possible that nectin-2 engages HHV-6B gB in multiple conformational states, which may allow for greater spatial flexibility than a rigid postfusion interaction. Further structural and functional studies will be required to comprehensively resolve these interactions. We hope our explanation addresses your concern

13) Line 769 – the Uniprot accession number is wrong.

**Response:** Thank you for your careful review. The incorrect Uniprot accession number (P36350) has now been corrected to the correct P36320.

14) ExtData Fig. 8b – the band for the purified V-set-Fc protein runs quite low, as I would expect the size of the monomer to be ~70 kDa. Is that normal?

**Response:** The nectin-2 V-set-Fc construct used in our study includes residues 1-158 of the V-set domain fused to an Fc region, with a predicted molecular weight of ~43 kDa. Considering potential glycosylation, the observed band ranging from 40 to 55 kDa in our Western blot is consistent with this expected size. For reference, the amino acid sequence of our construct is as follows: MARAAALLPSRSPPTPLLWPLLLLLLLETGAQDVRVQVLPEVRGQLGGTVELPCHLLPPVPGLYISLVTWQRPDAPANHQNVAAFHPKMGPSFPSPKPGSERLSFVSAKQSTGQDTEAELQDATLALHGLTVEDEGNYTCEFATFPKGSVRGMTWLRVGSEPKSCDKTHTCPPCPAPELLGGPSVFLFPPKPKDTLMISRTPEVTCVVVDVSHEDPEVKFNWYVDGVEVHNAKTKPREEQYNSTYRVVSVLTVLHQDWLNGKEYKCKVSNKALPAPIEKTISKAKGQPREPQVYTLPPSREEMTKNQVSLTCLVKGFYPSDIAVEWESNGQPENNYKTTPPVLDSDGSFFLYSKLTVDKSRWQQGNVFSCSpVMHEALHNHYTQKSLSLSPGK*

We appreciate your careful review and hope this explanation clarifies the observed molecular weight.

15) At the end of the discussion the authors claim that their findings will inform future efforts to develop vaccines. This claim, but also implications regarding the vulnerability of gB to neutralising antibodies should be discussed in light of recent findings by Sponholtz et. al, where they found that ‘No gB variants (pre or postfusion) in this study consistently elicited complement-independent neutralizing antibodies in mice.’ (doi.org/10.1073/pnas.2404250121)

**Response:** Thank you for this valuable suggestion. We have now incorporated a discussion on the findings of Sponholtz et al., as this study represents an important advancement in the field and is highly relevant to our work (Sponholtz et al., 2024)^12^. Their observation that prefusion-stabilized HCMV gB did not elicit higher neutralizing antibody titers than postfusion gB challenges the assumption that prefusion gB is inherently superior for vaccine development. This also underscores the potential immunogenic value of postfusion gB and support the consideration of both gB conformations in vaccine design. We appreciate this insightful recommendation, and we have revised our discussion to incorporate these insights, as shown below:

**Discussion:**

Additionally, vaccines incorporating gB as an immunogen for HSV, HCMV, and EBV have entered clinical trials. Notably, previous vaccine candidates have typically used recombinant postfusion gB ectodomains due to the instability of the prefusion conformation, and it has long been assumed that prefusion gB would elicit superior neutralizing responses. However, a recent study on HCMV gB successfully stabilized its prefusion conformation through introducing mutations but found it did not induce higher neutralizing antibody titers in mice compared to postfusion gB, suggesting that both pre- and postfusion gB conformations warrant consideration for vaccine design.

**Reference**

1	Lin, E. & Spear, P. G. Random linker-insertion mutagenesis to identify functional domains of herpes simplex virus type 1 glycoprotein B. *Proceedings of the National Academy of Sciences* **104**, 13140-13145 (2007).

2	Jean, F. *et al.* A protein-based therapeutic for human cytomegalovirus infection. *Proceedings of the National Academy of Sciences* **97**, 2864-2869 (2000).

3	Mahmoud, N. F. *et al.* Cytoplasmic tail domain of glycoprotein B is essential for HHV-6 infection. *Virology* **490**, 1-5 (2016).

4	Ogawa, H. *et al.* Nectin-2 acts as a viral entry mediated Molecule that binds to human herpesvirus 6B glycoprotein B. *Viruses* **14**, 160 (2022).

5	Oliver, S. L. *et al.* The N-terminus of varicella-zoster virus glycoprotein B has a functional role in fusion. *PLoS Pathogens* **17**, e1008961 (2021).

6	McCallum, M. & Veesler, D. Computational design of prefusion-stabilized Herpesvirus gB trimers. *bioRxiv* (2024).

7	Wakata, A. *et al.* The Neutralizing Linear Epitope of Human Herpesvirus 6A Glycoprotein B Does Not Affect Virus Infectivity. *Journal of Virology* **92** (2018). <https://doi.org/10.1128/jvi.02074-17>

8	Wakata, A. *et al.* The Combination of gQ1 and gQ2 in Human Herpesvirus 6A and 6B Regulates the Viral Tetramer Function for Their Receptor Recognition. *Journal of Virology* **95** (2021). <https://doi.org/10.1128/jvi.01638-20>

9	Zhong, L. *et al.* Targeting herpesvirus entry complex and fusogen glycoproteins with prophylactic and therapeutic agents. *Trends in Microbiology* **31**, 788-804 (2023).

10	Stampfer, S. D., Lou, H., Cohen, G. H., Eisenberg, R. J. & Heldwein, E. E. Structural basis of local, pH-dependent conformational changes in glycoprotein B from herpes simplex virus type 1. *Journal of virology* **84**, 12924-12933 (2010).

11	Cooper, R. S., Georgieva, E. R., Borbat, P. P., Freed, J. H. & Heldwein, E. E. Structural basis for membrane anchoring and fusion regulation of the herpes simplex virus fusogen gB. *Nature Structural & Molecular Biology* **25**, 416-+ (2018). <https://doi.org/10.1038/s41594-018-0060-6>

12	Sponholtz, M. R. *et al.* Structure- based design of a soluble human cytomegalovirus glycoprotein B antigen stabilized in a prefusion- like conformation. *Proceedings of the National Academy of Sciences of the United States of America* **121** (2024). <https://doi.org/10.1073/pnas.2404250121>
