# Supplementary material for: Human herpesvirus 6B glycoprotein B postfusion structure, vulnerability mapping, and receptor recognition
Source: PLoS Pathog. 2025 Jul 9;21(7):e1013300. doi: 10.1371/journal.ppat.1013300 (PMC12240383; doi:10.1371/journal.ppat.1013300)
Supplement: Attachment — Submitted filename: Response_to_Reviewers_auresp_2.docx [file ppat.1013300.s013.docx]

**Additional Editor Comments :**
1. Address the remaining concerns of reviewer 3.

**Response**: We have carefully addressed the remaining comments raised by Reviewer 3 in the point-by-point response and incorporated the corresponding revisions in the manuscript and figures as appropriate.

2. Incorporate important functional data currently shown in the Supplement into the main figures, to address the remaining concerns of reviewer 1. For example, Figures S7, S8, S5, and, potentially, other Supplemental figures.
**Response**: As recommended, we have moved key functional datasets from the Supplement into the main manuscript figures to address the remaining concerns of Reviewer 1. Specifically:

- Panels a, c, and d from former Supplementary Fig. S5 (SDS-PAGE analyses related to furin cleavage efficiency and *in vitro* cleavage assay) have been appended to the original Figure 2a, b.
- Supplementary Fig. 7b displaying footprints of neutralizing antibodies has been incorporated into Figure 3 to complement the structural presentation of epitope mapping.
- The immunoprecipitation (IP) results from former Supplementary Fig. 8c have been integrated into Figure 4, complementing the structural modeling of the gB–nectin-2 interaction.
- Additionally, the SDS-PAGE analysis of HHV-6B gB furin site variants (previously presented as Response Fig. 5)—which demonstrates increased cleavage of chimeric constructs carrying EBV or VZV furin motifs—has now been included in Extended Data Fig. 5a to better support the interpretation of furin processing.

We appreciate this suggestion, which has helped to better highlight the functional relevance of our findings within the main figures.

3. Add labels to all the figures so that the reader can follow them without having to go back and forth between the figure and the legend. For example, for each structural models, make sure to indicate whether it is obtained experimentally or predicted in silico. For SEC traces, indicate protein construct, and so on.
**Response**: We have carefully revised figures to ensure that critical annotations are directly embedded within the figure panels. Specifically, for SEC traces (Supplementary Fig. 1a and Fig. 8a, b), we have annotated each trace with the corresponding protein construct used. For structural models in Fig. 4 (now as Fig. 5), we have explicitly indicated within the panels whether each model is experimentally resolved or predicted in silico.

**Reviewers' Comments:**

Reviewer's Responses to Questions

**Part I - Summary**

Reviewer #1: As per my original review there is very little functional data included in this manuscript, a concern shared by myself and reviewer 3. There is a structure of HV6 gB and quite a bit of in silico modelling which is hypothesis generating but these are largely untested. There is some additional functional data included in the revision but it is all buried in the supplement. It might be suitable for publication if this were included in the main manuscript but I feel that there is not a significant advance here beyond the structure.

**Response**: We thank the reviewer for the critical assessment. As you pointed out, functional validation is relatively limited in the current study. This is in part due to technical constraints, such as the absence of available neutralizing antibodies specific to HHV-6B gB and the challenge of establishing a robust cell–cell fusion assay, which makes systematic functional assessment of mutant constructs difficult. Nonetheless, we sought to support the structural and computational findings through complementary experiments, including *in vitro* furin cleavage assays, biolayer interferometry (BLI), and immunoprecipitation-based interaction validation.

In response to your helpful suggestion, we have now moved these functional datasets from the Supplementary Figures into the main manuscript to improve accessibility and emphasize their relevance. Specifically, Supplementary Fig. 5a, c, d, 7b, and 8c—related to furin cleavage efficiency, neutralizing antibody footprints and gB–nectin-2 interactions, respectively—have been incorporated into the main manuscript as updated Figure. 2, 4, and 5, respectively. We appreciate your feedback, which has strengthened the clarity and presentation of the study.

Reviewer #2: The authors did a commendable job in revising the manuscript per the reviewer comments. The manuscript is a valuable contribution to the literature.

**Response**: We sincerely thank you for your positive evaluation and encouraging comments. We are grateful for your thoughtful feedback throughout the review process, which has helped us improve the clarity and overall quality of the manuscript.

Reviewer #3: During the review process Xie et al. put a lot of effort to address all the issues raised by the reviewers. Unfortunately, the functional relevance of the furin cleavage site was not determined. Nevertheless, I think this work and the structural information on HHV-6B gB it provides are relevant and will be interesting for the field of herpes virology. There are still minor things that should be addressed.

**Response**: We are grateful for your engagement and constructive input throughout the review process. We have addressed your points in detail in our responses below.

**Part III – Minor Issues: Editorial and Data Presentation Modifications**

Reviewer #3: 1) Comparison of the experimental structure and the alphafold model
It is good that the authors followed my suggestion and performed the alphafold prediction. I think it would be useful to also show the RMSD between the prediction and the experimental structure. The RMSD values could also be incorporated in the table in Extended Data Fig. 6b. These results should also be mentioned in the manuscript, as it is important to show the quality of the prediction, but even more so to also highlight details that cannot be predicted by Alphafold yet. In Extended Data Fig. 3a the differences could be highlighted with zoom-ins on the structural alignment.

**Response**: Thank you for this thoughtful follow-up suggestion. In response, we quantified the structural similarity between the AlphaFold3-predicted model and our cryo-EM structure by calculating the root-mean-square deviation (RMSD) across the entire ectodomain and for individual domains. These RMSD values have been incorporated directly into Supplementary Fig. 3a, to facilitate side-by-side interpretation with the structural alignment. To better illustrate the differences, we have also added zoomed-in panels to highlight regions such as the DII–DIII linker and fusion loops (Response Fig. 1). In the manuscript, we have updated the Results section to describe these RMSD comparisons and emphasize that while the overall AlphaFold3 model aligns well with the experimental structure, several flexible or functionally relevant regions, particularly the furin site linker and fusion loops, deviate significantly, as shown below. We appreciate your suggestion, which has helped us provide a more complete comparison between the predicted and experimentally determined structures.

**Result:**

The structure of HHV-6B gB reveals a conserved overall architecture that is consistent with that of other herpesvirus gB in postfusion conformation. We further compared our experimentally determined structure with an AlphaFold3-predicted model of HHV-6B gB. The predicted model aligned well with the cryo-EM structure (RMSD = 1.386 Å), capturing the domain organization and overall fold (Supplementary Fig. 3a). Notable differences were observed in the linker between DII and DIII containing the furin cleavage site, which exhibited a shifted position compared to the experimental structure, and in the DI fusion loops, which adopted different orientations between the predicted and experimental models (Supplementary Fig. 3a).

**Response Fig. 1|** AlphaFold3-predicted structure colored by pLDDT confidence score (left) and structural alignment of the AlphaFold3 model with the cryo-EM structure (right). Zoomed-in views of selected regions highlight structural differences, including the DII–DIII linker containing the furin cleavage site and the fusion loop region. The table summarizes the RMSD values (in Å) between the predicted and resolved structures for individual domains and the ectodomain.

2) Functional relevance of the furin cleavage site
I appreciate the effort the authors put in, in order to functionally characterise the furin cleavage during fusion. As the authors pointed out, using HCMV gB enabled fusion, meaning the assay per se is working. This also means that more efficient cleavage of HHV-6B gB (with the EBV / VZV furin cleavage site substitutions) does not increase fusion efficiency of gB. This point might be mentioned in the paper.

**Response**: Thank you for this follow-up suggestion. As noted, we observed clear fusion activity when replacing HHV-6B gB with HCMV gB, confirming that the assay platform itself is functional. However, fusion signals mediated by HHV-6B gB were indistinguishable from vector control, indicating that we were unable to establish a functional HHV-6B gB-mediated cell-cell fusion assay. Therefore, we believe the current system does not support a reliable quantitative comparison between wild-type and furin-site–modified HHV-6B gB constructs. For this reason, we opted not to include the fusion assay results in the revised manuscript. Nevertheless, we now include the SDS-PAGE–based analysis of the EBV- and VZV-site–substituted HHV-6B gB ectodomains (previously shown as Response Fig. 5a), now presented as Supplementary Fig. 5b in the manuscript. This analysis supports that the native cleavage motif contributes to the low furin susceptibility of HHV-6B gB. The corresponding description has been added to the Results section, as shown below. We are grateful for this helpful suggestion, which has helped clarify the limitations of our current system.

**Result:**

We further compared our experimentally determined structure with an AlphaFold3-predicted model of HHV-6B gB. The predicted model aligned well with the cryo-EM structure, with a root-mean-square deviation (RMSD) of 1.386 Å, capturing the domain organization and overall fold (Supplementary Fig. 3a). Notable differences were observed in the linker between DII and DIII containing the furin cleavage site, which exhibited a shifted position compared to the experimental structure, and in the DI fusion loops, which adopted different orientations between the predicted and experimental models (Supplementary Fig. 3a).

3) a-Helix X in HSV-1 gB
This more a comment than a suggestion. I appreciate the authors efforts to clarify the structural differences in the region corresponding to HSV-1 gB residues 462–477. It is indeed interesting that in HHV-6B gB the region forms an extended loop instead of a helix. Also, as far as I can see, the Alphafold prediction shows a loop rather than a helix, further supporting this finding.
**Response**: We thank the reviewer for this encouraging comment. We are glad that the analysis of this region and the consistency between the experimental structure and AlphaFold3 prediction were found to be informative.

4) Nectin-2 gB interaction
I thank the authors for the explanation about the potential interaction. It would be helpful to indicate in Figures 4b and c, where the rest of Nectin-2 would be located. And also, to mention in the text the reasons why a receptor interaction with gB in this position might not be obstructed by the membrane.

**Response**: Thank you for this helpful suggestion. In response, we have updated Fig. 5b to schematically indicate the approximate spatial arrangement of full-length nectin-2 relative to the HHV-6B gB interaction site (Response Fig. 2). This illustration highlights how the unresolved membrane-proximal region (MPR) of gB and the additional Ig-like domains of nectin-2 likely extend the spacing between the two membranes, mitigating potential steric hindrance. We have also added a brief explanation in the Results section to address this point.

**Result:**

Notably, the predicted V-set binding site on the external lateral side remains largely similar and accessible in the prefusion conformation, suggesting the potential of nectin-2 to interact with HHV-6B gB in both prefusion and postfusion forms. And while DI lies relatively close to the membrane in both conformations, the unresolved membrane proximal region of gB and the additional Ig-like domains in full-length nectin-2 likely provide additional spatial extension on both sides, allowing receptor access without membrane obstruction (Fig. 5b). Furthermore, given the dynamic nature of gB transitions, it remains possible that nectin-2 engages HHV-6B gB in multiple conformational states, which may allow for greater spatial flexibility for interaction.

**Response Fig. 2|** Predicted interaction between the nectin-2 V-set domain and HHV-6B gB. The interaction between the nectin-2 V-set domain and HHV-6B gB was predicted via AlphaFold2 based on the resolved HHV-6B gB structure from this study and the reported V-set structure (PDB: 3R0N). The pLDDT confidence score for the prediction is indicated by the scale bar. The central panel displays a cartoon representation of the predicted interaction, with the HHV-6B gB protomer colored as in Fig. 1b. A zoomed-in view highlights the predicted interacting residues, shown as sticks. To illustrate the spatial context of this interaction relative to the membranes, the approximate positions of the unresolved membrane-proximal region (MPR) of gB, the additional Ig-like C2 domains of full-length nectin-2, and the viral and host membranes are schematically indicated.
